# Radiological measurement of pelvic fractures using a pelvic deformity measurement software program

**DOI:** 10.1186/s13018-020-1558-2

**Published:** 2020-01-31

**Authors:** Shuwei Zhang, Gongzi Zhang, Ye Peng, Xiang Wang, Peifu Tang, Lihai Zhang

**Affiliations:** Department of Orthopedics, Chinese PLA General Hospital, No. 28 Fuxing Rd. Haidian District, Beijing, 100853 People’s Republic of China

**Keywords:** Pelvic fractures, 3D reconstruction, Measurement software program, Method and formulas, Pelvic closed reduction

## Abstract

**Background:**

It is difficult for the surgeon to measure pelvic displacement in the closed reduction operation for unstable pelvic fracture. We therefore developed a pelvic deformity measurement software program based on standardized radiographs. The objectives of the present study were to evaluate the inter-observer reliability of the program for measuring specific fracture types on preoperative pelvic films and to assess the validity of the measurement software program by comparing it with a gold standard.

**Methods:**

Twenty-five patients diagnosed with AO/OTA type B or C pelvic fractures with the unilateral pelvis fractured and dislocated were included in this study. Four separate observers repeatedly determined the translational and rotational patterns and outcomes using the software program and hand measurement, and calculated the displacement using computed tomography (CT) coupled with a three-dimensional (3D) CT model. The validity of the measurement software was calculated by assessing the consistency between the software measurements and the gold standard. Additionally, inter-observer reliability was assessed for the software. The software was also applied in preliminary clinical practice for closed reduction procedures.

**Results:**

The overall inter-observer reliabilities of the software program, CT coupled with 3D reconstruction, and hand measurements were high, with kappa values of 0.956, 0.958, and 0.853, respectively. The software showed validity similar to that of CT coupled with 3D reconstruction (0.939 vs. 0.969), and better than that of hand measurement (0.939 vs. 0.858). A preliminary clinical application demonstrated that the software is effective for guiding closed reduction of pelvic fractures.

**Conclusions:**

Our newly established pelvic deformity measurement program is a reliable and accurate tool for analyzing pelvic displacement patterns and can be used for guidance of closed reduction and planning of the reduction pathway.

**Level of evidence:**

III

## Background

Unstable complex pelvic fractures are challenging for orthopedic trauma surgeons, having high morbidity, mortality, and healthcare costs, and they are increasing in incidence [1–3]. Postoperative complications of pelvic fracture, which are related to suboptimal bone healing and various degrees of inability to transfer load from the trunk to the lower limbs, render the restoration and operative stabilization of the pelvic ring an imperative goal [4].

Conventional open operative management of unstable pelvic fractures (type B and C) can achieve direct visualization, anatomical reduction, and rigid fixation. However, this approach (“Kocher-Langenbeck” or “Stoppa”) traditionally requires substantial surgical exposure of the internal structure deep in the pelvis. Such exposure can cause severe complications, including major blood vessel or nerve damage, denervation or devascularization of the abductors, excessive hemorrhage, high incidence of infection, wound-healing problems, and heterotopic ossification [5–8]. Closed reduction and percutaneous internal fixation, which can reduce bleeding, decrease surgical time and radiation exposure, and accelerate postoperative recovery, appear to be a solution [9–11]. However, successful closed reduction of a three-dimensional (3D) multidirectional pelvic deformity is difficult to achieve [12, 13]. Additionally, inadequate reduction may lead to residual displacement, which can cause adverse long-term outcomes [14, 15]. McLaren et al. [16] and Lindahl et al. [17] reported that a residual deformity greater than 10 mm was a sign of poor prognosis. Lindahl and Hirvensalo [18] further concluded that unsatisfactory reduction or loss of reduction displayed a statistically significant correlation with unsatisfactory functional results. Residual deformity of the posterior pelvic ring has been shown to be associated with persistent pain [19–21]. Hence, anatomical reduction or near anatomical reduction is an essential task for pelvic ring reduction. Additionally, the achievement of anatomical reduction through traditional closed reduction of pelvic fractures is difficult, requiring highly experienced surgeons for manual manipulation, and anatomical reduction is often subjectively verified by surgeons [22].

We previously established a computer-aided method based on intraoperative computed tomography (CT) images and their 3D reconstruction to assist in pelvic closed reduction [23]. However, this method required the surgeon to master computer-aided design software. Additionally, an intraoperative CT system was required in the operation room. Although CT is regarded as the gold standard for true displacement, it is time-consuming and involves ionizing radiation. It is therefore difficult to monitor pelvic displacement in real time, and operating room requires high conditions. Moreover, emergent radiographs often fail to demonstrate the total amount of displacement and therefore cannot truly reflect the injury that took place in a traumatic event. Gardner et al. found that a significant amount of recoil occurred passively in the pelvic mode in the absence of a deforming force, and pointed out that the initial images may underestimate the degree of pelvic displacement [24]. Furthermore, transferring and positioning patients may also change the pelvic deformity pattern. Thus, a reevaluation of the pelvic deformity in the operating room with a C-arm after general anesthesia is of benefit for closed reduction. Therefore, a reliable and accurate measurement tool based on intraoperative roentgenography is necessary for the reevaluation and anatomical reduction of pelvic fractures.

We therefore developed a new method to measure radiographic displacement of unstable pelvic ring fractures [25]. Our method was shown to be reliable, and it limited measurement error. We further developed a software tool based on our previous method for unilateral pelvic fractures, and this tool allowed complex calculation steps to be completed in a few clicks, resulting in appropriate balancing of data integrity and reliability. The objectives of the present study were as follows: (1) to evaluate inter-observer reliability for measuring the displacement pattern of unilateral unstable pelvic fractures (type B and type C) on preoperative pelvic films, and (2) to assess the validity of the measurement software program by comparing it with a gold standard measurement and hand measurement.

## Methods

### Design and setting

This study involved medical imaging investigations and was approved by the ethics committee of the Chinese PLA General Hospital. After obtaining informed patient consent, data on the demographic characteristics of 25 patients with unilateral pelvic fractures who were admitted to the Department of Orthopaedics and Trauma at the Chinese PLA General Hospital between March 2014 and March 2015 was prospectively collected.

All 25 patients met our inclusion criteria: (1) a relative stable general condition, (2) had unilateral unstable pelvic fractures suitable for closed reduction and minimally invasive fixation, (3) they were willing to participate in the study and 8- to 12-month follow-up. Patients with severe associated injuries that were treated in the same operation as the pelvic fractures and patients that were unsuitable for closed reduction and minimally invasive fixation were excluded.

We also collected preoperative CT images in Digital Imaging and Communications in Medicine (DICOM) format. All CT scans were performed using a Somatom Sensation open CT system (Siemens AG, Erlangen, Germany) with a slice thickness of 1.5 mm. The CT data (DICOM) were imported into Amira software (Visage Imaging GmbH, Berlin, Germany) to reconstruct and simulate the standard inlet, outlet, and AP views of the pelvis.

### Definition of the pelvis-specific coordinate system

**Fig. 1:**
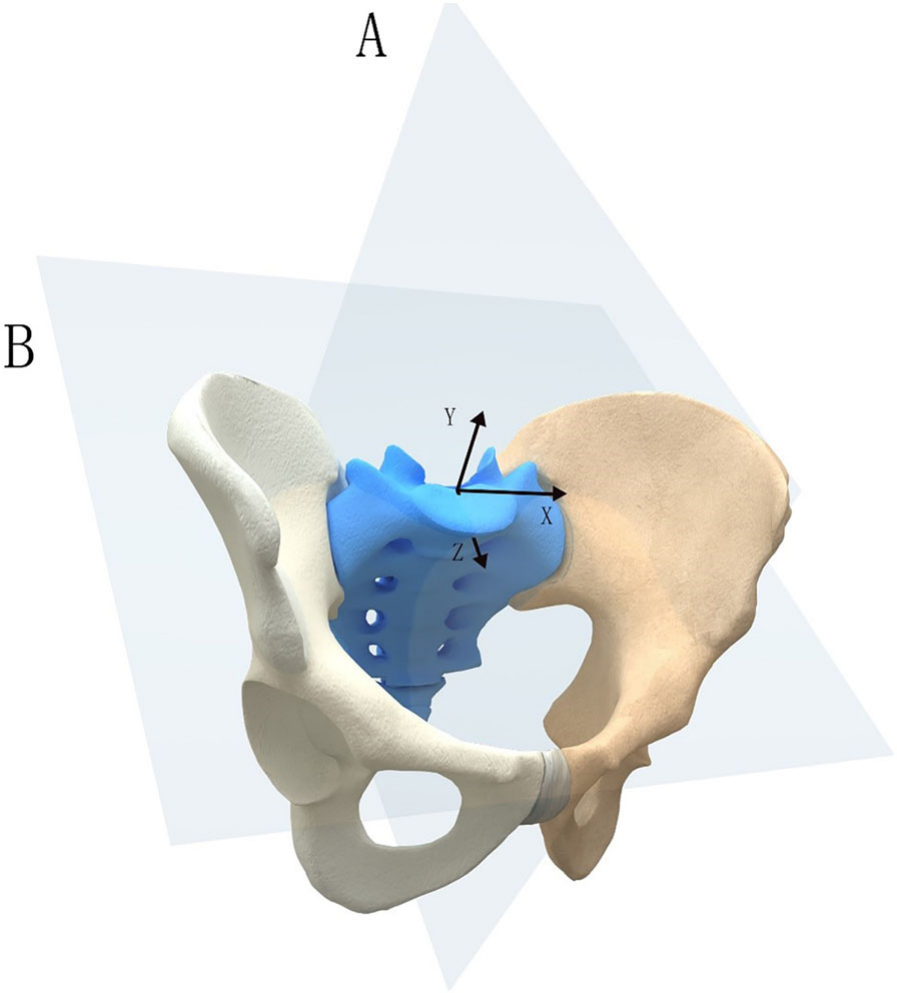
The specific coordinate system of the pelvis in three dimensions

The pelvis has a complex spatial structure and a deep body, making it hard to describe a pelvic deformity directly. Our measurement method depended on symmetry and utilized the intact or minimally injured contralateral side as a reference. To better describe the displacement types of pelvic fractures and to provide standardized steps for measurement, we established a pelvis-specific coordinate system in three dimensions, as shown in Fig. 1. We defined the standard mutually orthogonal inlet and outlet radiographs as *X*/*Z* and *X*/*Y* planes, respectively, and the sagittal plane perpendicular to the *X*/*Z* and *X*/*Y* planes as the *Y*/*Z* plane. The origin of the coordinate system was defined as the trailing edge of the end plate.

### Statistical analysis

The inter-observer reliability and validity between the four separate observers were assessed using Cohen’s kappa value. According to the criteria of Landis and Koch, the kappa value can be divided into six ranges representing the strength of agreement and degree of consistency, with a kappa value < 0 indicating no agreement, 0.00–0.20 slight agreement, 0.21–0.40 fair agreement, 0.41–0.60 moderate agreement, 0.61–0.80 substantial agreement, and 0.81–1.00 almost perfect agreement. The kappa values and their 95% coefficient intervals were calculated using SAS 9.1.3 software (SAS Institute Inc., Cary, NC, USA).

### Method and formulas for measuring displacement

Deformity of the hemipelvis is classified into three mutually perpendicular axes (*X*/*Y*/*Z*) and two movement types (translational and rotational), resulting in six basic displacement patterns.

As described in our previous study [25], several representative anatomical landmarks were first selected (Table 1). On anterior-posterior radiographs, the anterior superior iliac spine (ASIS) and ischial tubercle were marked to detect the pelvic rotational movement around the *Y*-axis in the sagittal plane. On inlet radiographs, the anterior SI joint (iliac side), ASIS, and center of the sacral endplate were selected to measure the transverse movement along the *Z*- and *X*-axes and rotational displacement around the *Y*-axis. On outlet radiographs, the superior point of the iliac wing, ASIS, and ischial tubercle were used to determine the transverse movement along the *Y*-axis and rotational movement around the *Z*-axis.

**Table 1:** The representative anatomic landmarks selected for the measurement of the displacement of the hemipelvis

Radiographs	Representative anatomic landmarks
AP view	Anterior superior iliac spine
	Ischial tuberosity
Outlet view	Superior point of iliac wing
	Ischial tuberosity
	ASIS
	Center of sacral endplate
Inlet view	ASIS
	Anterior SI joint (iliac side)
	Center of sacral endplate

*ASIS* anterior superior iliac spine

The vertical and horizontal lengths were measured on equal standard radiographs, which can represent the actual displacement of the pelvis, and the findings were then compared with constants. The specific measurement steps have been described in a previous study [25].

## Measurement software description

### Software development

The pelvic deformity measurement software was written using C# and provides semi-automated descriptions of six displacement patterns of pelvic fractures. Each radiograph imported into the software is reconstructed using Amira software and scanned digitally as a Digital Imaging and Communications in Medicine (DICOM) format file. Displacement measurements on radiographs are taken following the instructions and prompts provided on the interface. Once a radiograph view is selected, a series of prompts and diagrammatic sketches pointing out anatomical landmarks instruct the user to select a variety of landmarks in the inputted radiographs. The software then performs the necessary calculation process to automatically determine the pattern of pelvic fractures, including the following three sections: inlet plane, outlet plane, and AP plane (Additional file 1).

### Reliability and validity study

In this study, 25 sets of preoperative radiographs (anteroposterior [AP], inlet, and outlet views) simulated by Amira, CT scan data, and a 3D-CT reconstruction model of pelvic fracture patients were measured by four independent observers, including two senior orthopedic residents (LHZ and YP) and two fellowship-trained orthopedic surgeons (JXZ and ZGZ). The simulated radiographs of the pelvic fractures, which were standard and could represent actual distance, were imported into the software in sequence and measured directly in the software. The CT data of each patient were collected retrospectively through the Patient Archiving and Communications System and were reconstructed into 3D models by an individual observer (XYS) using Mimics software (Materialise, Haasrode, Belgium).

All analyses were conducted in a blinded fashion, 1 week apart, and in a random order and independent manner. The four observers were asked to record the pelvic deformity pattern on the three orthogonal planes after each measurement, and to not change their answer after the measurement. The displacement was first determined according to hand measurement results and software measurement results based on the radiographs in sequence. The four observers independently analyzed the CT data and its 3D reconstruction, then discussed the findings and reached an agreement. The consensus was used as the gold standard for true deformity results.

The inter-observer reliability of the software measurement system was assessed using the kappa statistic with 95% confidence intervals. For each movement pattern, the validity was evaluated according to the consistency levels across the gold standard, the CT coupled with its 3D reconstruction, the results of the measurement software, and the hand measurements. Inter-observer reliability was determined for all the abovementioned orthopedics. If the observers’ judgments of a movement pattern were inconsistent, they were considered as inconsistencies among observers. The consistency level for each movement pattern was determined according to the consistency of each observer’s results between the software program, hand measurement, and 3D CT reconstruction. The four observers were required to become familiar with and master the software method, such that they could complete the measurement independently.

### Preliminary clinical application for pelvic closed reduction: a representative case describing the whole surgical procedure

**Fig. 2:**
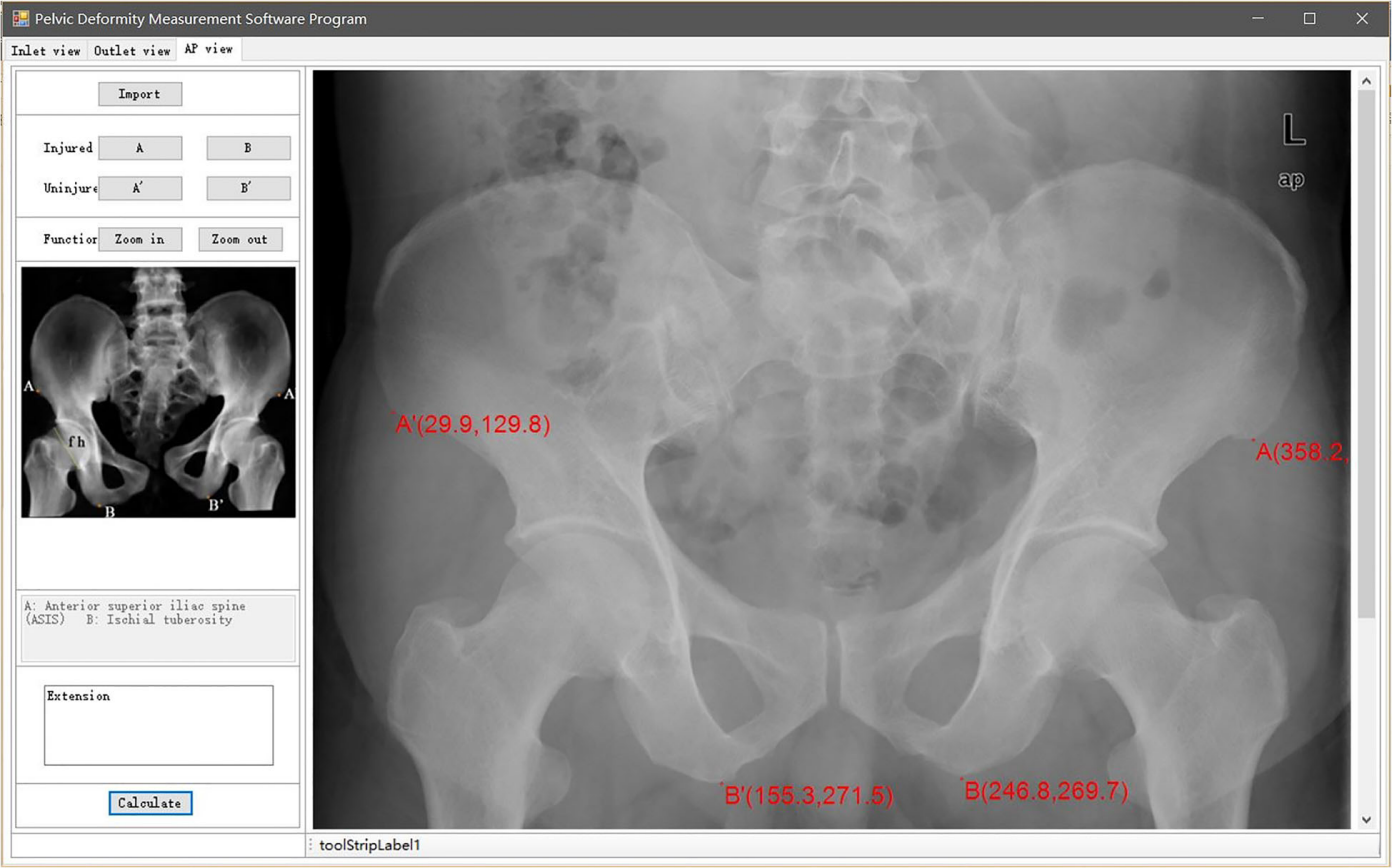
Measurement results in the anteroposterior view

**Fig. 3:**
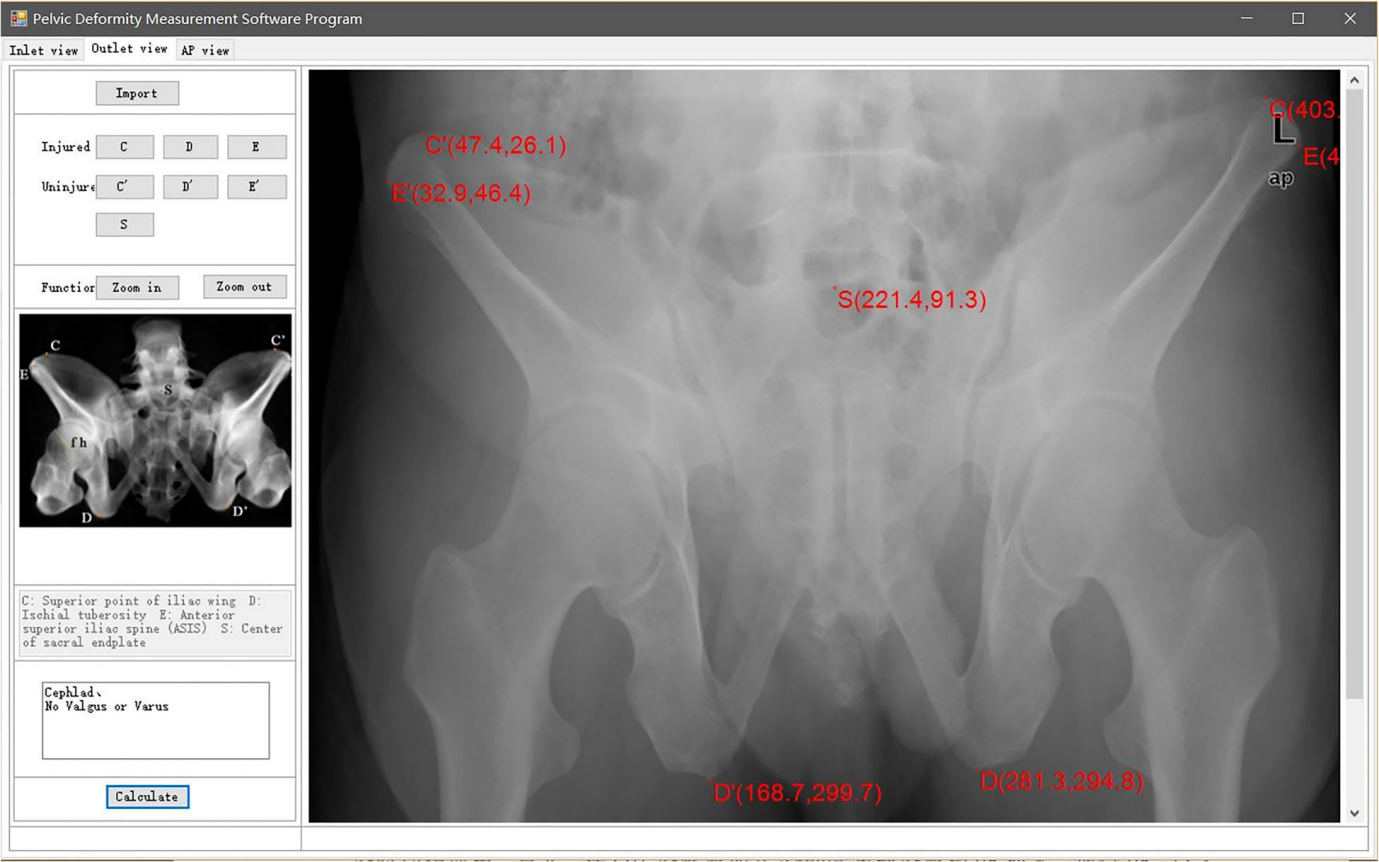
Measurement results in the outlet view

**Fig. 4:**
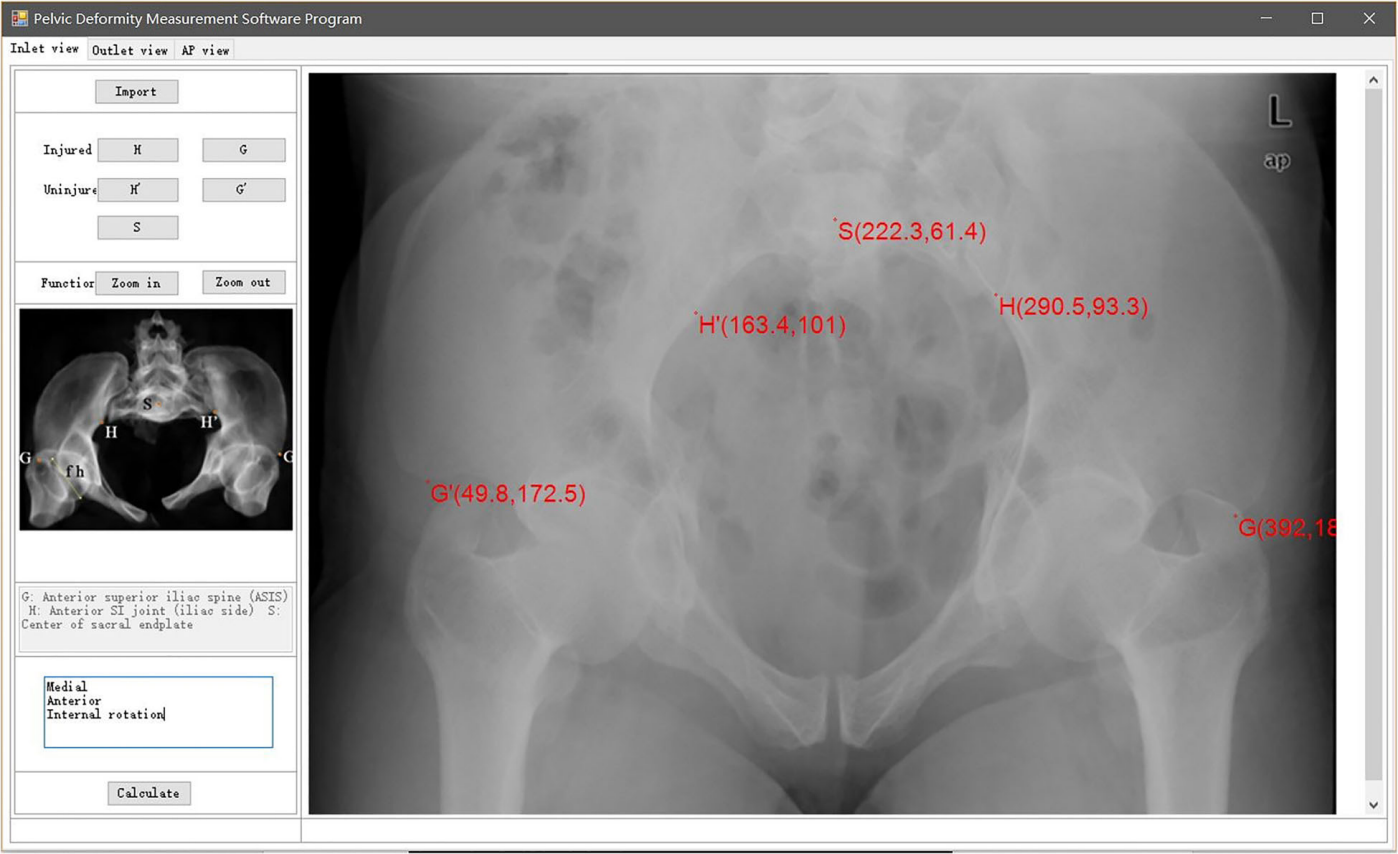
Measurement results in the inlet view

A 47-year-old man fell from a height of 6 m. He was initially taken to a nearby hospital complaining of lumbosacral pain, and he was diagnosed with a pelvic fracture. A CT scan showed a left hemipelvic fracture (Young–Burgess type LCP; Tile type B2). Nine days after injury when his general condition was stable, he underwent closed reduction and minimally invasive fixation. He received general anesthesia and was placed in the supine position. We reevaluated the pelvic displacement using a C-arm and obtained AP, inlet, and outlet radiographs in DICOM format, and imported the images into the pelvic deformity measurement software program. The results demonstrated the following specific pelvic patterns: extension in the AP view (Fig. 2), cephalad displacement in the outlet view (Fig. 3), and medial anterior displacement and internal rotation in the inlet view (Fig. 4). The calculation took only 2 min.

The calculation results provided the surgeons with not only a comprehensive impression of the pelvic deformity in 3D, but also information on the specific displacement types in each plane. With a better understanding of the pelvic deformity characteristics, the surgeons planned the reduction pathway with the assistance of a previously assembled reduction frame, which was a six-degrees of freedom (DOF) remote center of motion (RCM) mechanism composed of three one-DOF RCM mechanisms [23]. In our experience, the more convenient approach is to first correct the translational displacement and then correct the rotation. We corrected the cephalad displacement using left distal femoral traction and then pushed and laterally moved the LC-II screw to correct the medial anterior displacement in the inlet view. Finally, the extension in the sagittal plane and internal rotation in the inlet view were corrected by sliding the LC-II screw on the side arcs of the frame. Intraoperative radiographs showed that anatomical reduction was obtained, and closed reduction was accomplished. Two percutaneous iliosacral screws (S1 and S2) were appropriately placed to hold the reduction, and the reduction frame was removed.

At his 8-month follow-up appointment, the patient displayed normal gait and minimal discomfort, and he had returned to his previous job. Radiographs showed no loss of reduction.

## Results

### Reliability and validity of the results

The study population included 25 patients (14 men and 11 women) with a mean age of 41.04 ± 13.85 years at injury. The patients had AO/OTA (Arbeitsgemeinschaftfür Osteosynthesefragen/Orthopaedic Trauma Association) type C (unstable, complete disruption of posterior arch) and type B (partially stable) unilateral pelvic fractures.

The assessments of pelvic displacement based on the software, CT data (coupled with 3D reconstruction), and hand measurement showed almost perfect agreement and high reliability, with kappa values greater than 0.8 and a lower 95% confidence interval greater than 0.75 (Table 2). Table 3 demonstrates the reliability of the translational and rotational measurements of the three measurement methods, and shows higher inter-observer agreement for the translational displacements than for the rotational displacements (CT coupled with 3D reconstruction, 0.964 vs. 0.949; software program, 0.974 vs. 0.936; hand measurement, 0.894 vs. 0.802, respectively). The consistency between each measurement method and the gold standard is shown in Table 4. The software program and CT data (coupled with 3D reconstruction) had similar effectiveness for analyzing pelvic displacement patterns, with both having kappa values greater than 0.9 (0.939 and 0.969, respectively).

**Table 2:** Inter-observer analysis for overall displacement

Measurement method	Kappa value	95% CI
Software program	0.957	0.929–0.984
CT coupled with 3D reconstruction	0.958	0.930–0.987
Hand measurement	0.853	0.823–0.883

*CT* computed tomography, *3D* three-dimensional, *CI* confidence interval

**Table 3:** Inter-observer analysis for translational and rotational displacement

	Measurement method	Kappa value	95% CI
Translational	Software program	0.974	0.926–1.023
	CT coupled with 3D reconstruction	0.964	0.915–1.013
	Hand measurement	0.894	0.844–0.945
Rotational	Software program	0.936	0.890–0.982
	CT coupled with 3D reconstruction	0.949	0.902–0.996
	Hand measurement	0.802	0.752–0.853

*CT* computed tomography, *3D* three-dimensional, *CI* confidence interval

**Table 4:** Validity analysis for hemipelvic displacement

Measurement method	Kappa value	95% CI
Software program	0.939	0.905–0.973
CT coupled with 3D reconstruction	0.969	0.934–1.003
Hand measurement	0.858	0.823–0.894

*CT* computed tomography, *3D* three-dimensional, *CI* confidence interval

Overall, the software program had the highest reliability score for translational displacement, and CT coupled with 3D reconstruction had the highest reliability score for rotational displacement and overall displacement, and scored best in the validity analysis for hemipelvic displacement.

## Discussion

### Main findings

It is difficult for the operator to measure pelvic displacement in closed reduction operations for unstable pelvic fractures. We therefore developed a software program to measure pelvic deformity on standardized radiographs. The overall inter-observer reliabilities of the software program, CT coupled with 3D reconstruction, and hand measurements were high, with kappa values of 0.956, 0.958, and 0.853, respectively. The software showed validity similar to that obtained with CT coupled with 3D reconstruction (0.939 vs. 0.969), and better than that obtained with hand measurement (0.939 vs. 0.858). Preliminary clinical applications demonstrated that the software was effective for guiding closed reduction of pelvic fractures.

### Comparisons with other studies

Pelvic fractures represent approximately 8% of blunt trauma injuries and are associated with high mortality and disability [26]. Deformity of the pelvic ring often leads to pelvic asymmetry, pelvic instability, and subsequent complications, such as residual pain and movement disorders, which are associated with poor patient outcomes [27, 28].

Traditional pelvic classification systems for preoperative planning and reduction pathway guidance for unstable pelvic fractures are no longer sufficient for clinical and research purposes [29], and a thorough understanding of pelvic deformity is important for further evaluation, closed reduction, and safe screw and external fixator pin placement. Currently, specific types of pelvic fractures are assessed with the aid of roentgenography and CT scans. Viegas et al. reported that 3D-CT reconstruction techniques had a volumetric validity of 94% and a linear validity of 97% [30]. Nystrom et al. demonstrated that CT and its resulting computer-reconstructed radiographs (CRRs) showed good validity and reliability, especially for assessing rotational displacement [31]. Although CT is regarded as the gold standard for true displacement, it is associated with high cost and excessive radiation exposure [31, 32]. Hence, some orthopedic surgeons attempt to analyze pelvic displacement using radiographs, which are conventional and commonly used in clinical settings.

Lefaivre et al. collected pre- and postoperative radiographs from 25 patients with OTA B or C pelvic ring disruption, assessed the reliability using three representative methods, and then reviewed 31 manuscripts from 28 authors published between 1990 and 2009 involving the radiographic measurement of pelvic fractures [33, 34]. Although there was an advantage with using three preoperative films, the absolute displacement method of Lefaivre et al. [35] showed poor reliability in contrast with the moderate agreement of the impression of the observers. The cross measurement method of Keshishyan et al. [36] based only on the AP view showed excellent agreement, and the inlet/outlet ratio measurement method described by Sagi et al. [37] showed almost perfect agreement on preoperative radiographs. It was shown that a high number of views, choices, and steps available to an observer were associated with poor agreement [34]. However, as the pelvis is a complex 3D structure, it is difficult to describe pelvic displacement completely from one or two views. Comprehensiveness and reliability appear to form a paradox; therefore, although measurement methods for pelvic deformity have been described by many authors, there is still no widely accepted standardized method for describing the complete information of pelvic fractures with high reliability and validity. The pelvic deformity measurement software program appears to be a solution for the abovementioned paradox.

The inner measurement method was shown to be reliable and valid in our previous study [25]. However, it appears to be slightly difficult to master, and there may be errors from hand measurement. Therefore, we created a measurement software program that can semi-automatically measure the specific pelvic displacement patterns from radiographic planes (inlet, outlet, and AP view), and evaluated its reliability, validity, and preliminary clinical application status. Although the reliability of the software was slightly lower than that of CT coupled with 3D reconstruction, the software showed near perfect agreement in all views and all movement patterns and was more reliable than hand measurement. The validity of the software was assessed according to its consistency with the gold standard, which was almost perfect (0.939, 95% confidence interval 0.905–0.973). The likely reasons for the high reliability and validity of the software were well-defined landmarks, a standardized semi-automated measurement procedure, and simple operation steps, which substantially reduced human-induced measurement error. The software well resolved the paradox between comprehensiveness and reliability. The simple operation and short learning period make the software more suitable for young surgeons with limited experience of pelvic fracture. The four observers in our study took a mean of 55 ± 9 min to grasp the software and less than 10 min to analyze pelvic deformity using the three X-ray planes after practicing for a mean of 7 ± 1.4 times.

Previously, reduction of pelvic displacement depended mostly on a surgeon’s experience. In our preliminary clinical application, the surgeons took full advantage of the software and found an evident decline in the reduction time. Compared with hand measurement, the software can provide the surgeon with a more comprehensive and reliable impression of the pelvic displacement, which is very helpful for guiding closed reduction and planning a reduction pathway. Compared with CT and 3D reconstruction, the software depends on real-time fluoroscopy, which is less time-consuming.

The pelvic deformity measurement software program has several advantages. First, it comprehensively describes the spatial pelvic displacement with translational and rotational patterns in three mutually orthogonal axes based on X-ray images, and thereby covers all possible types of pelvic deformity in three dimensions. Second, it shows good reliability and validity when compared with the gold standard and has obvious advantages over hand measurement. Third, it turns a sophisticated measurement procedure into a simple measurement approach with a few clicks in the radiograph planes. It requires less operating room equipment, avoids the high cost and large amount of radiation of CT, and can be used to evaluate pelvic displacement in real time during the closed reduction process. Vertical and transverse distances are both automatically calculated once the landmarks are selected. Fourth, considering the more ubiquitous presence of fluoroscopes in the operating room and the reduced radiation exposure in comparison with CT, our software is a good choice for the measurement of pelvic displacement.

The present study has some limitations. First, our software can only assess unilateral pelvic fractures with AO/OTA classifications of type B or C. Second, the study sample was small; however, we will expand the number of clinical cases in the future. Third, our software fails to calculate the exact extent of the deformity. Nonetheless, the surgeon can take advantage of the software program for comprehensive preoperative planning and overall reduction pathway guidance. Surgeons should use real-time fluoroscopy to observe specific details and the extent of closed reduction and percutaneous fixation. The next stage in the development plan for the software is to add the capability to measure the translational distance and rotational angle.

## Conclusions

Our newly developed pelvic deformity measurement software program is a comprehensive, reliable, and accurate tool for analyzing the displacement patterns of unilateral pelvic fractures with AO/OTA classifications of type B or C, and can reduce human-induced error. It showed similar efficacy to that of CT coupled with 3D reconstruction and can be utilized for guidance in closed reduction and planning of the reduction pathway.

## Supplementary information

**Additional file 1.** The pelvic deformity measurement software program. (RAR 929 kb)

## Data Availability

Not applicable

## Abbreviations

3D: Three-dimensional
AO/OTA: Arbeitsgemeinschaftfür Osteosynthesefragen/Orthopaedic Trauma Association
ASIS: Anterior superior iliac spine
CT: Computed tomography
DICOM: Digital Imaging and Communications in Medicine
DOF: Degrees of freedom
RCM: Remote center of motion
